# Peter Paul Rickham: the Liverpool neonatal surgery unit 1953

**DOI:** 10.1007/s00383-024-05910-x

**Published:** 2024-11-28

**Authors:** Paul D. Losty

**Affiliations:** 1 Institute of Systems Molecular and Integrative Biology, University of Liverpool, Liverpool, UK; 2 Department of Paediatric Surgery, Alder Hey Children’s Hospital, Liverpool, UK; 3 Department of Paediatric Surgery, Ramathibodi Hospital, Mahidol University, Bangkok, Thailand

**Keywords:** Peter Paul Rickham, Isabella Forshall, Herbert Johnston, Jackson Rees, Neonatal surgery, Anaesthesia, Paediatric surgery, Urology, History, PPR Gold Medal

## Abstract

This article highlights the evolution, birth and legacy of the world’s first neonatal surgical unit established at Alder Hey Children’s Hospital Liverpool in 1953. Peter Paul Rickham, a creative pioneering and innovative surgeon, is credited here as a major driving force that helped shape and progress the modern day development of neonatal surgery. Rickham’s vision was realised by studying neonatal surgical disorders and the mortality rate of congenital anomalies in Mersey Region while working as a young senior registrar with Isabella Forshall. Rickham defined the extent of the problem(s) and set to work as a newly appointed consultant a vision for improving outcomes with the creation and establishment of a preeminent world leading neonatal surgical unit. Surgeons from all over the world travelled to work, learn and train with Rickham and the paediatric surgical staff team at Alder Hey in the subsequent years to follow. Neonatal anaesthesia greatly advanced by Jackson Rees a colleague working in Liverpool with Rickham allowed huge success to flourish—‘the impossible became possible’. The neonatal surgical unit in Liverpool became the benchmark and prototype for units to develop around the world immediately resulting in improvement in the survival of newborn infants undergoing surgery from 22 to 74%. Rickham’s contributions to neonatal and paediatric surgery are truly remarkable. Alder Hey hosts an international symposium and special dinner with Rickham family members as VIP guests in its calendar of events. A symposium highlight is the Rickham Lecture with the PPR Gold Medal awarded to an international renowned leader in the field of paediatric surgery and the surgical sciences.

As the Second World War (WW II) was ending in 1945, Britain like much of the rest of the world was in a recovery cycle journey. Deprivation and social inequality in the major cities of the UK was all too evident for most to see and the health of citizens a priority. The UK National Health Service (NHS) established in 1948 by a Labour Government—providing health care ‘free at point of delivery’—and available to all citizens through funding by taxation—sought to bring great hope to the nation [1]. Child health was a major medico-political problem also at this time and of course this included the newborn infant. Deaths from congenital anomalies required some scrutiny and a young paediatric surgeon Peter Paul Rickham—having served in the Royal Army Medical Corps (RAMC) in WW II—arrived at Alder Hey Children’s Hospital Liverpool in April 1949 and set about investigating the state of the problem [2].

## Early history birth and development of the world’s first neonatal surgery unit in Liverpool

Rickham—writing in the Lancet—reported that during a 10-year period 1938–1947 from studying Liverpool Maternity Hospital records some 2% of 20,479 live births were associated with congenital anomalies and 12.6% of 475 neonatal deaths were directly resultant from congenital malformations. In England and Wales, during 1949, extent of the problem was even much greater with over 2056 babies less than 4 weeks of age dying of congenital malformations [2]. 

‘I found after my arrival only two neonates with the surgical condition congenital duodenal obstruction survived in Liverpool, both were operated upon by my senior colleague Isabella Forshall [3]. This disastrous state of affairs was not peculiar only to Liverpool. It was closely mirrored by centres all over Europe’ [4]—quoted Peter Rickham.

In May 1949, only after a short time when Rickham came to Liverpool, a female infant was born with oesophageal atresia [4, 5]. In 1944, Haight and Towsley in America had published the first early success with the condition [6]. In 1947, Richard Franklin working at the Hammersmith Hospital in London later reported the first two successful cases from the UK and Europe [7]. Thanks to superb anaesthesia and skills of Jackson Rees—a leading pioneer in neonatal anaesthesia—the baby girl having undergone the emergency operation undertaken by Rickham and Forshall thankfully survived with vigilant postoperative care cited as proving critical to the successful outcome [5, 8–10]. The baby—it is understood—was nursed ‘24/7 round the clock’ by Rickham and his medical colleagues Dr Robert Todd and Dr Allan Kirby until hospital discharge occurred on the 40th day postoperatively [4, 5].

‘I am convinced that this arrangement afterwards called by Professor Barry O’Donnell of Dublin Ireland as “the first neonatal intensive care department” was the reason the baby survived’—Rickham later stated. This index case marked the beginning of the birth of neonatal surgical intensive care as we now know it in the world today [4].

In May 1953, the world’s first neonatal surgical unit was inaugurated at Alder Hey Children’s Hospital Liverpool. The unit originally consisted of eight cots and two–six infant incubators with rooms available on the ward for nursing mothers crucial to assisting with care of their newborns [4, 11]. Beyond the City of Liverpool, the later rapid development of neonatal transport services to follow across North West England and Wales led to all surgical neonates in a wide geographic regional area covering a population exceeding 3.5 million being cared for at Alder Hey. With over 200 index cases/annum being treated at Alder Hey its surgical, nursing and medical paediatric staff were now working at the very forefront of ‘neonatal surgery coming of age’ [4, 11].

**Fig. 1:**
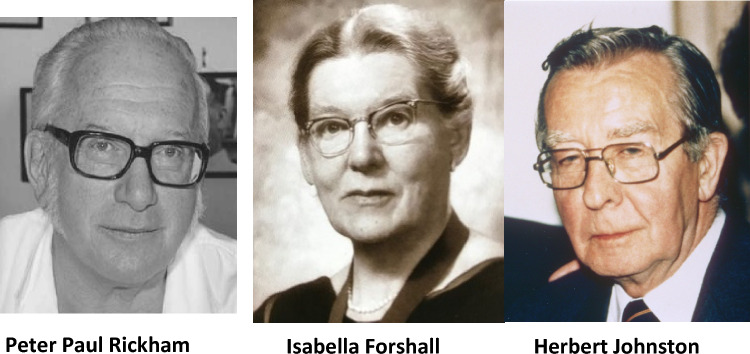
Peter Rickham, Isabella Forshall and Herbert Johnson—Consultant Staff Surgeons “A Trilogy of Giants”

In 1960, Rickham and Forshall later published a landmark article in the Lancet titled—‘Experience of A Neonatal Surgery Unit—The First Six Years’ showing spectacular outcomes as result of growing skills expertise and the concentration of neonatal surgery at Alder Hey as their recipe for success [12]. Some 663 newborns during this study time period had undergone surgical operations with an astounding 75% survival rate. Staff surgeon numbers had at this time period also increased from two consultant surgeons Peter Paul Rickham and Isabella Forshall later being joined by Herbert Johnston from Belfast Northern Ireland as the third partner in the Department of Paediatric Surgery at Alder Hey in 1956 (Fig. 1). Surgical workload at Alder Hey in the months and years to follow expanded momentously not only with neonatal surgery index case load activity but also general paediatric surgery and urology and wider referrals arriving to the hospital from all over North West Region of England, Wales and Isle of Man.

## Surgeons travel the globe to train with Rickham and team at Alder Hey, Liverpool

**Fig. 2:**
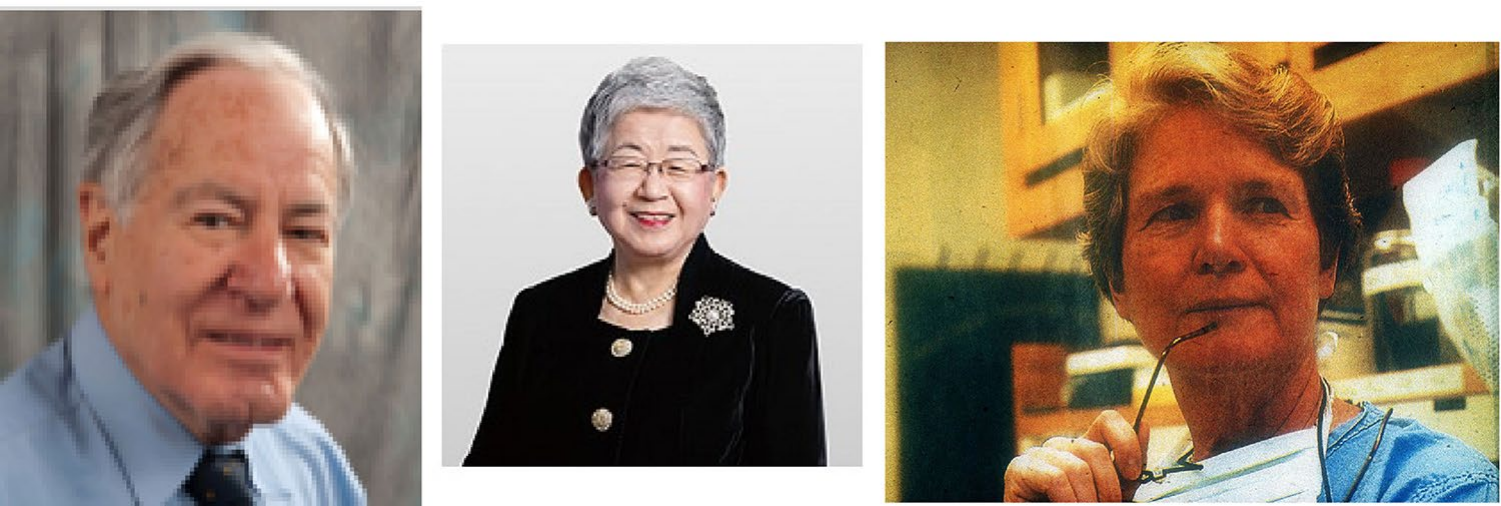
Lewis Spitz—Nuffield Professor of Paediatric Surgery (1979–2004) Great Ormond Street Children’s Hospital, UCL London, UK; Professor Sachiyo Suita—Kyushu University Hospital, Fukuoka Japan; Patricia K. Donahoe MD—Surgeon-in-Chief of Pediatric Surgery & Director of Pediatric Surgical Research Laboratories at Massachusetts General Hospital Harvard Medical School Boston USA

Young surgeons from many world continents particularly those from America, Japan and South Africa eagerly travelled to Liverpool to train with Rickham and the surgical staff team at Alder Hey. Many of these surgeons later advanced to became world leaders in paediatric surgery in their native countries having been individually trained and mentored by Rickham (Fig. 2). Rickham purposely designed the Alder Hey surgical training programme based on that which he had carefully studied and observed as so successful working with Robert Gross at Boston Children’s Hospital USA. The list of surgical residents during these years is too many and extensive to report in full though some notable key names amongst others include—Lewis Spitz from South Africa—a Smith and Nephew Fellow—who later became the Nuffield Professor and Chair of Paediatric Surgery at Great Ormond Street Hospital London in 1979; Sachiyo Suita inducted as the first female Professor of Paediatric Surgery in Japan at Kyushu University Hospital Fukuoka and Patricia K Donahoe from Boston—Professor of Pediatric Surgery at Massachusetts General Hospital Harvard Medical School, USA (Fig. 4).

## Innovation and research championed by Rickham

Peter Rickham had a deeply enquiring and inventive mind. He is credited with many innovations in paediatric surgery. The Rickham reservoir was incorporated into the critical design of ventriculoperitoneal shunt systems for hydrocephalus allowing accurate dynamic pressure measurement to test for shunt malfunctioning and direct access to the ventricular system for emergent decompression including CSF sampling in suspected infection(s) [13]. A custom designed newborn and infant operating table, incubator development(s) and a wide portfolio of surgical research in the basic sciences and clinical paediatric surgery is all evident from Rickham’s prolific career journey [14, 15]. Rickham’s MS thesis and some of his foremost research publications were on physiology and surgical metabolism including intravenous nutrition. In 1969, Peter Rickham and Herbert Johnston published the landmark textbook titled ‘Neonatal Surgery’ which was to become—quote—the ‘bible of neonatal surgery’ according to Professor Lewis Spitz who read it from cover to cover and it was widely used also by paediatric surgeons throughout the globe with record sales [16, 17].

Rickham was one of the early surgeon pioneers who advocated aggressive timely treatment of bladder and cloacal exstrophy malformations and he also played a leading role working in Zurich Switzerland (vide infra) in promoting minimally invasive surgery alongside Stephen L. Gans (USA) also in the 1970s [18, 19]. Rickham had a deep interest in medical ethics publishing widely in this field championing the treatment of congenital anomalies [20].

## Zurich—1971 and beyond ...

**Fig. 3:**
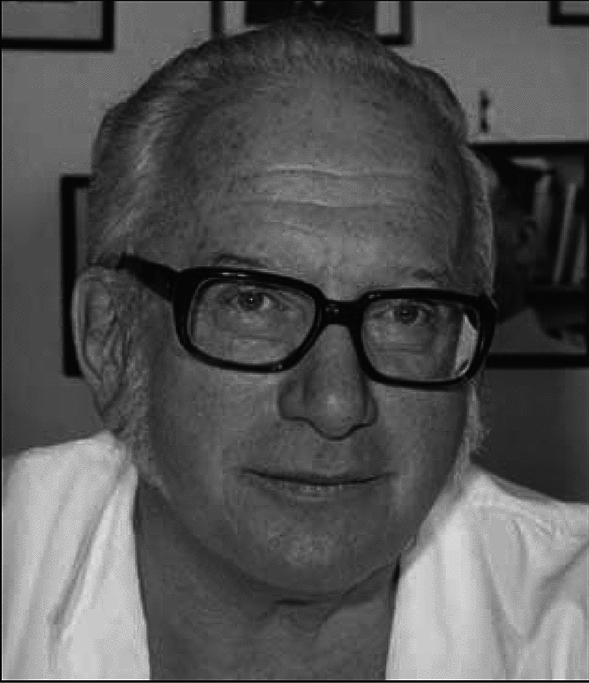
Photograph portrait of Peter Paul Rickham—an original copy is displayed in the main lecture theatre at University Children’s Hospital Zurich Switzerland

In 1971, having accomplished so much at Alder Hey in Liverpool, Peter Rickham later moved to Zurich Switzerland as the new Chief of Paediatric Surgery at the University Children’s Hospital to a ‘dream job’ it is understood where his success in innovation and research continued with ever greater enthusiasm to successfully build another world class department together with Urs Stauffer and his other surgical colleagues until his retirement in 1983. A framed photograph of Rickham to this very day hangs on the wall in the main lecture theatre at the hospital in honour and recognition of his great service to the University Children’s Hospital in Zurich (Fig. 3).

## Rickham legacy and symposia

Peter Rickham was one of the early founding members of the British Association of Paediatric Surgeons in 1953 later serving as BAPS President in 1967–1968. He was also a founding member of the European Union of Paediatric Surgeons, the Society for Research in Hydrocephalus and Myelomeningocele and an Editor for the Journal of Pediatric Surgery. He was awarded many honours and tributes in a long distinguished professional career travelling across the world as a popular key note lecturer and visiting professor. Distinctions amongst which there are too many to list here included the Denis Browne Gold Medal—BAPS, Commander’s Cross—Germany, Legion d’ Honneur—France—the highest award that can be bestowed on a civilian by the French republic and the W.E. Ladd Medal from the Surgical Section American Academy of Pediatrics (AAP). Peter Rickham published extensively with more than 200 original papers in leading scientific journals including being the Editor-in-Chief of several major textbooks and seminal monographs. Rickham had a notably distinguished career in WW II taking part in the Normandy invasion and also serving in the Far East. He reached the rank of Major in the Royal Army Medical Corps. Interests outside medicine included classical music, skiing, painting and hiking [21, 22].

**Fig. 4:**
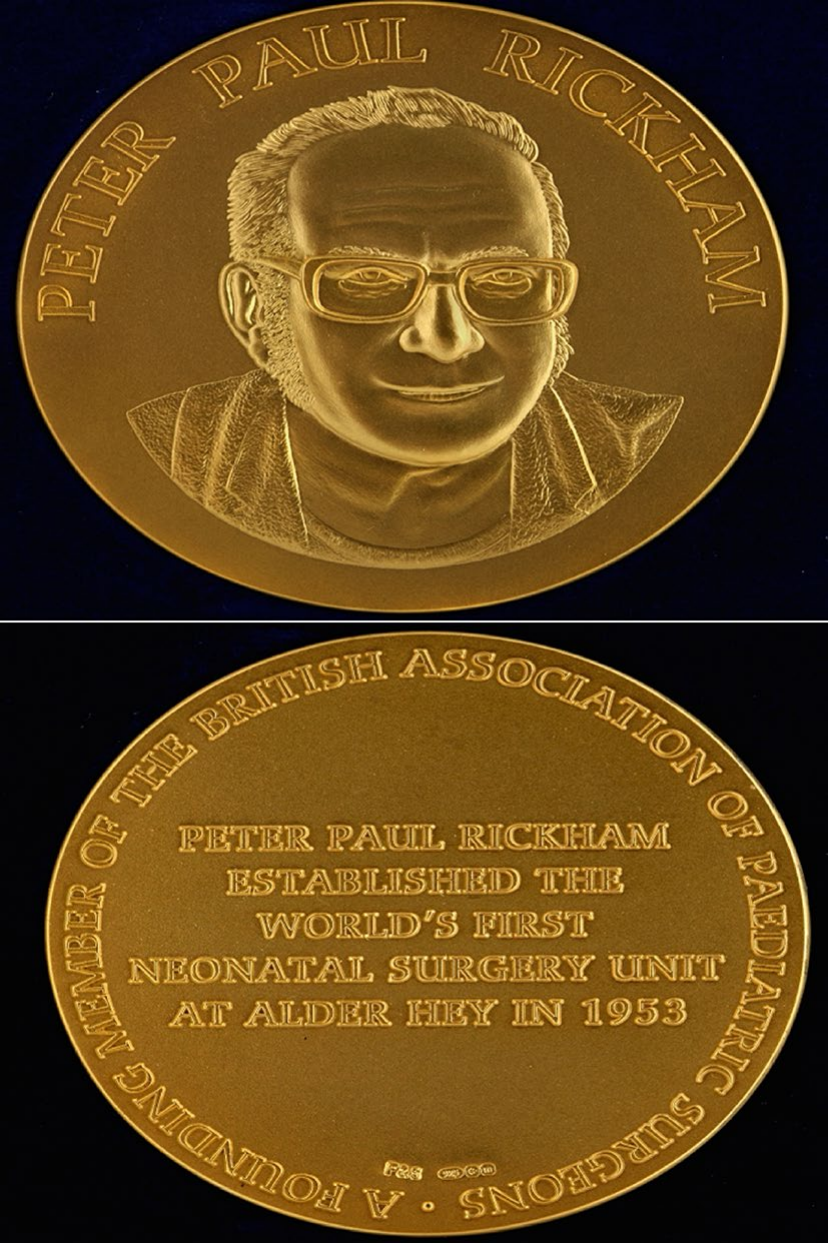
PPR Gold Medal—awarded to key note lecturer at the Rickham international paediatric surgical symposium meeting(s). Medal on front face—Peter Paul Rickham; reverse side—engraving states—‘A founding member of the British Association of Paediatric Surgeons (BAPS); established the world’s first neonatal surgery unit at Alder Hey in 1953’

In the late 1980s, a ‘benefactor’ according to Professor David Lloyd made a mystery financial donation to the Department of Paediatric Surgery at Alder Hey to help host an international surgical symposia meeting at the hospital. The meetings it was suggested by the anonymous benefactor could perhaps be themed ‘Rickham Symposium’!! Alder Hey celebrates Peter Paul Rickham with a symposium held on a regular calendar basis on the university hospital campus attended by many leading world personalities together with a designated keynote lecturer—by special invitation—tasked to deliver the Rickham Lecture. A PPR gold medal in recognition is awarded to the Rickham Lecturer (Fig. 4). Rickham family members are always in attendance as special VIP’s at the symposia event with an evening dinner and speeches delivered from guests to mark this very special celebratory occasion.

## Peter Paul Rickham—a biography summary

Peter Paul Rickham (1917–2003) was born in Berlin Germany in 1917 and graduated from Medical School at Cambridge University, UK. In his early medical training and surgical residency days, he worked at St Bartholomew’s Hospital, London, and then progressed to Great Ormond Street Hospital London serving under Denis Browne moving on then to Alder Hey Children’s Hospital Liverpool as senior registrar joining Isabella Forshall in 1949. Rickham subsequently spent a year overseas at Boston Children’s Hospital with Robert Gross and Charles Everett Koop at Children’s Hospital of Philadelphia USA as a Harkness travelling fellow scholar before being finally appointed consultant paediatric surgeon at Alder Hey in 1952. He later became the Director of Academic Paediatric Surgical Studies at Alder Hey in 1965 where he continued in post with tireless energy driving forward clinical and basic science research in Liverpool. In 1971, Peter Rickham was appointed Professor of Paediatric Surgery at the University Children’s Hospital in Zurich Switzerland where he remained until his retirement in 1983.

Peter Paul Rickham was predeceased by his first wife Elizabeth to whom he was married for over some 60 years. He remarried Lyn Chang who nursed him through a final long illness sadly dying on November 17th 2003 following a stroke. Peter Rickham is survived by Lyn and three children from his first marriage—a son—David, two daughters—Susan and Mary-Anne and five grandchildren [21, 22].

Peter Paul Rickham’s life-time accomplishments in Neonatal and Paediatric Surgery are truly remarkable and legendary.

## Data Availability

No datasets were generated or analysed during the current study.
